# Integrating social and ecological considerations in floodplain relocation and restoration programs

**DOI:** 10.1007/s42532-023-00152-y

**Published:** 2023-05-04

**Authors:** Linda Shi, Shanasia Sylman, Carri Hulet, Rebecca Morgenstern Brenner, Amelia Greiner Safi, Paul Corsi

**Affiliations:** 1 Department of City and Regional Planning, Cornell University, 213 Sibley Hall, Ithaca, NY USA; 2 CH Consulting, Medford, MA USA; 3 Brooks School of Public Policy, Cornell University, Ithaca, NY USA; 4 Department of Public and Ecosystems Health, Cornell University, Ithaca, NY USA; 5 SUNY Upstate Medical University, Syracuse, NY USA

**Keywords:** Flood relocation, Buyout policy, Socio-ecological systems, Climate adaptation, Environmental justice

## Abstract

In the United States, most floodplain relocation (or buyout) programs focus on moving homeowners, then deal separately with what happens with the land afterward. These programs typically divide processes for relocation planning, engagement, funding, and implementation from those related to post-buyout land management and restoration. The structural and operational conditions that lead to this separation of roles and responsibilities miss out on opportunities to create more synergistic socio-ecological strategies that may produce healthier outcomes for both people and the environment. In other domains, research shows that healthy people and healthy environments can co-create each other through more virtuous cycles. In this perspective essay, we argue that we can better create such virtuous cycles in floodplain relocation programs by integrally considering social and ecological components. Such efforts can encourage more people to decide to relocate, thereby creating more contiguous places to restore. They can also empower more residents to help steward these sites, an action that in turn helps heal and strengthen flood-affected communities. These arguments, while particular to the United States, have resonance for floodplain management and land use planning worldwide.

## Introduction

After Hurricane Sandy struck New York City in 2012, the city removed nearly 700 homes across Staten Island, the Rockaways, and other waterfront neighborhoods through government-facilitated relocation, or buyout, programs (Feldman 2022). Ten years later, the City organized a series of virtual conversations on “What Happens with the Land?” to discuss what to do with the now-vacant low-lying coastal properties. This example of how relocation activities is segmented from those deciding the fate of the land is typical of efforts nationwide. Usually, buyout programs focus on negotiating with households to relocate them and transfer property title. Later, the local entity now holding the property title becomes responsible for deciding what happens with that land and how to maintain it. Each of the pre- and post-buyout components raises their own thorny questions of equity, efficiency, and effectiveness, which a growing body of scholarship engages. In this perspective essay, we add to these debates by asking: what might we gain by better connecting socially-oriented relocation processes with ecologically-oriented land management processes? How might their integration help practitioners working on relocation and restoration overcome the challenges particular to each phase and accelerate attention to the land post-buyout?

Local and regional governments increasingly face these questions as climate change exacerbates the intensity and frequency of storms devastating homes along coasts and waterways. Worldwide, 283 million people were displaced from climatological events between 2008 and 2020 (IDMC 2021). In the United States, over one million people are displaced by climatological disasters each year, the sixth highest rate of internal displacement globally (Perls 2020, p. 512). Yet, even the most prominent U.S. government program to relocate households through a buyout program (the Federal Emergency Management Agency’s Hazard Mitigation Grant Program (FEMA HMGP)) has cumulatively removed only 40,000-some properties over the last thirty years (Mach et al. 2019, p. 2).

Relocation permanently removes people and buildings from high-risk areas and can be a more effective long-term solution compared to seawalls or levees in reducing flood risk (Siders et al. 2019). But often, these efforts focus on immediate flood risk reduction and rehousing residents without more holistic attention to the well-being of relocatees, remaining residents, and ecological systems (Balachandran et al. 2022; Ehrenfeucht and Nelson 2022; Iuchi 2014). Both people who move away and those who stay behind can face greater stress from financial burdens, as well as loss of mental health and social cohesion (Barile et al. 2020; McGhee et al. 2020). A land use study of buyouts acquired between 1990 and 2000 found that 40% transitioned to recreational or park facilities, 40% remained as vacant or parking lots, and only 7.5% were ecologically restored (Zavar and Hagelman 2016, pp. 365–8).

In the future, how can relocation programs deliver better social and ecological results as they scale up to meet the need under climate change? We argue that more integration between buyout processes and post-buyout land management practices can help produce synergistic socio-ecological outcomes for both the environment and residents who either remain or relocate. This perspective reflects our experiences as scholars and practitioners who have worked with governments and communities on buyout and restoration processes. Collectively, we have engaged with buyout regulators, funders, elected leaders, managers, and staff in local, regional, state, and federal government agencies across the country. These people have worked in parks and public works departments, code enforcement, emergency management, watershed coalitions, utilities, flood control districts, and conservation commissions, among others. We have also engaged with residents who have either relocated or remained in-situ to learn how buyouts have affected their families and neighborhoods. While we focus on FEMA’s HMGP program, these concepts are also relevant to buyout programs funded by other federal agencies.

In the sections below, we first review research in diverse fields that show how community and ecological health can sustain each other in virtuous cycles to demonstrate the benefits of connecting socio-ecological systems. We then show how a leading buyout program in the United States segments the property purchasing process from floodplain management, housing relocation, community health, and ecological restoration. We argue that recognizing and investing in socio-ecological relationships can improve participation in buyouts, enhance stewardship of post-buyout sites, and build both environmental and community health. We conclude with broader policy changes requiring advocacy as well as suggested practices that practitioners can use within existing structural constraints.

## Human-nature relationships are multi-faceted and reinforcing

How do we define and conceive of the relationship between people and nature or, put another way, people and land? This fundamental question rests at the heart of adapting to climate impacts. In the Anglo-American legal context, this relationship between people and land is most often framed as property ownership, with all benefits ultimately reducible to a single characteristic: property value. However, this preoccupation with property value as the primary measure of human interactions with land limits our understanding of what we need to adapt to and recover from, such as land- or water-based livelihoods, ancestral and spiritual connections, and place-based social networks (Siders and Ajibade 2021; O’Donnell 2023, p. 6–7). O’Donnell (2023) pushes us to challenge our underlying assumptions and value systems in order to achieve transformative change, which also echoes Indigenous and other non-Western perspectives on climate change action (Whyte 2020; Yee et al. 2022). One starting point is recognizing that people’s relationship to the land is varied and multifaceted.

Studies from the fields of socio-ecological systems, water resource development, cultural landscape management, public health, environmental education, biodiversity conservation, and geography, among others, show that humans and nature shape each others’ well-being, whether in virtuous or vicious reinforcing cycles (Varis 1999, p. 599; Selman and Knight 2006; Tidball and Krasny 2011, pp. 4, 10–13; Morrison 2016, p. 11). Given interdependence between social and ecological functioning, impacting one can improve or degrade the other (Adger 2000, pp. 359–60). For example, increased impervious surfaces and structures in a floodplain can lead to diminished natural drainage, increased flooding, and recurring disruptions to everyday life. By contrast, nurturing both the social and ecological components of a community builds capacity to cope with crises by strengthening diverse and redundant alternatives (Folke et al. 2010). While ecological restoration has been known to improve human well-being, there is now growing recognition that restoration of both social and ecological components is better achieved through reparative processes (Fernández-Manjarrés et al. 2018, p. 409).

**Fig. 1:**
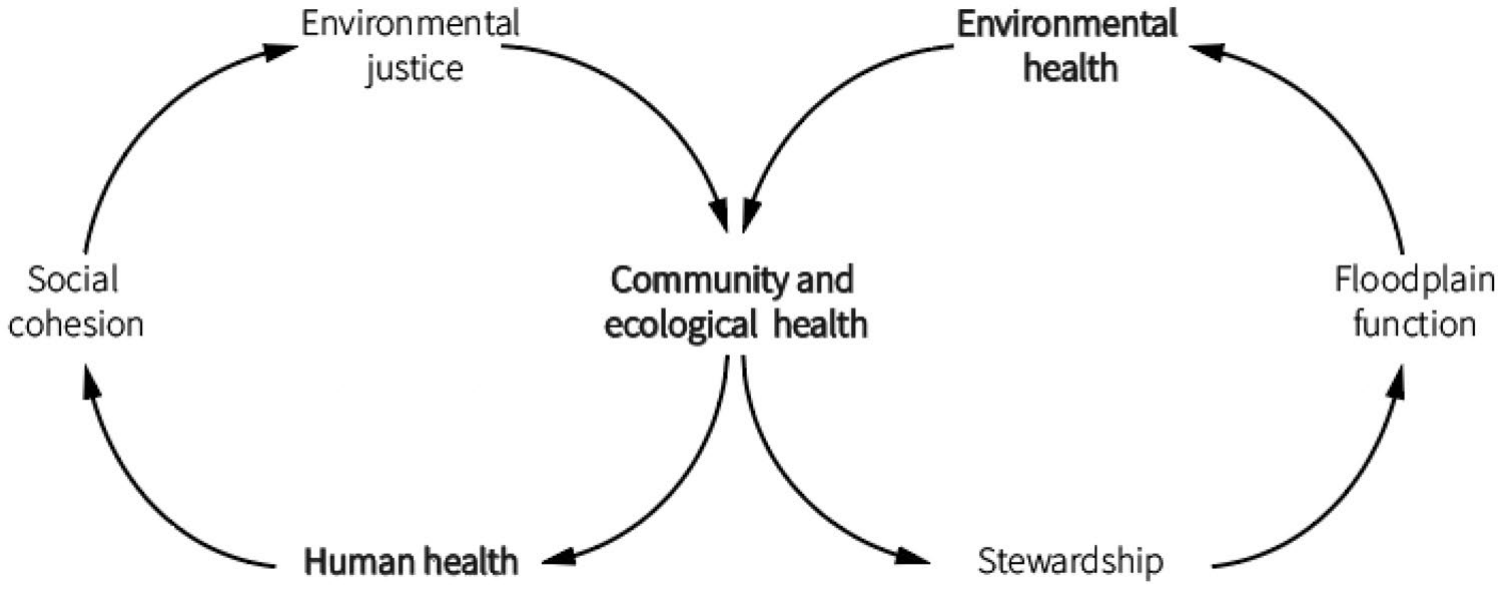
Benefits of Virtuous Socio-Ecological Relationships

Figure 1 shows how community and environmental health mutually reinforce one another. On the left, healthy environments contribute to human health, which in turn supports the formation of stronger social relations that can help remedy environmental injustices. On the right, healthy communities are more likely to engage in stewardship activities that help promote or restore floodplain function and healthy environments. People do not simply inhabit space or avoid harm by leaving a precarious environment. Rather, human health, identity, spirituality, culture, and self-efficacy derive at least in part from human interactions with nature. Environmental stewardship activities can improve people’s physical and mental health, as well as their sense of agency (Wolf et al. 2013, p. 29). Below, we expand on a few virtuous socio-ecological relationships that can improve post-disaster recovery outcomes.

### Quantity and quality of green spaces contribute to human health

Contact with nature is associated with diverse health benefits (Frumkin et al. 2017). A positive association exists between physical health, mental health, and the quantity of green spaces in human living environments, especially in urban areas (Ward Thompson et al. 2016; van den Berg et al. 2015, p. 813). Contact with nature also impacts social health (Jennings and Bamkole 2019; Maas et al. 2009, p. 593), culture (Milfont and Schultz 2016), and spirituality (Ferguson and Tamburello 2015; Heintzman 2009). Other health benefits include lowered risk of COVID-19 mortality with greater exposure to green spaces (Yang et al. 2022, p. 9). Greening cities by increasing the amount of green space has the potential to improve the health of its residents, contribute to local sustainability and placemaking (Dadvand and Nieuwenhuijsen 2019; Maller et al. 2009).

Both quantity and quality of green space can impact human health. While quality of green space does not have a shared definition, it encompasses several characteristics, such as natural features (e.g., land cover and vegetation mix), infrastructure and amenities (e.g., paved walkways and seating), maintenance and safety (e.g., absence of litter and presence of incivilities), some of which have demonstrated benefits to health (Nguyen et al. 2021). In addition to objective measures, the perceived quality of green spaces influences its accessibility and usability, thereby impacting human health. For example, Reid et al. (2022, p. 12–3) found that the perceived quality of a nearby green space could be associated with lower anxiety levels. This extends to the context of buyouts. Residents of post-buyout neighborhoods in Lexington, Kentucky shared with Zavar (2015, p. 88) that the lowered flood risk of the green spaces around them improved their overall well-being and reduced stress, while still acknowledging that the spaces could be further improved. It is important to recognize that green spaces offer many health benefits, but access to and enjoyment of these spaces are also shaped by race, class, and gender. While health inequalities can be potentially improved through increased exposure to nature, access to green space is still mediated through discriminatory practices and social exclusion (Seaman et al. 2010, p. 7).

### Environmental stewardship can improve ecological and social outcomes

The extent to which humans feel connected to and responsible for their surrounding environments also affects positive environmental behaviors and goals (Geng et al. 2015, p. 7; Mackay and Schmitt 2019, p. 7; Mayer and Frantz 2004, p. 512; Restall and Conrad 2015, p. 273). Positive environmental behaviors, such as environmental stewardship, can come from many motivations. There are intrinsic motivations, like values and ethics, and extrinsic motivations, like social reinforcement and economic benefits (Bennett et al. 2018, p. 602). For example, a survey of participants in urban agriculture reported multiple motivations, with “improving the environment” being the highest rated. The strongest positive impact of this was to their mood and physical well-being (Kirby et al. 2021, p. 5). Additionally, ecological restoration and its potential to create green jobs and economic opportunity can also influence environmental stewardship motivations (BenDor et al. 2015; Falxa-Raymond et al. 2013; Mansuy 2020). Whatever the driver, environmental action in turn contributes to a community’s social capital and adaptive capacity (Whitburn et al. 2019, p. 801).

Although acquired parcels often become the responsibility of local government after a buyout and may result in a patchwork distribution, there still are community management opportunities (e.g., gardens, pollinator habitats, small-scale green infrastructure, and pocket parks) on individual lots (Kihslinger and Salvesen 2018, p. 11). Kihslinger and Salvesen (p. 12) share the example of Happy Hill Community Garden in Rocky Mount, North Carolina, which was established on post-Hurricane Floyd buyout land. Taking action to manage local green spaces can help communities develop local knowledge and collaborations for other civic activities and capacity for learning (Pretty and Smith 2004). Case studies elsewhere have confirmed that positive environmental actions, such as organizing community gardens, can build social capital and be a springboard for citizens into broader social movements (Saldivar-Tanaka and Krasny 2004, p. 408). Such actions can also build the cohesion needed to respond to disaster impacts, and provide a form of economic support for those with limited opportunities (Hou 2017, p. 126). Thus, while the role of local stewards is often overlooked in land use management and planning, such forms of community involvement can be critically important for promoting and sustaining the landscapes we all depend on (Andersson et al. 2014, p. 445).

### Environmental justice has both social and ecological benefits

Buyouts have a close connection to environmental injustices—both historic (i.e., public housing built in flood-prone areas (Furman Center 2017)) and ongoing (who gets access to buyout resources (Siders and Keenan 2020)). Such injustices can contribute to vicious cycles that deteriorate both social and ecological systems. These injustices are due to systemic environmental racism in terms of where housing has been built and who is able to shape these policies (Bullard 2001, p. 160–1; Pulido 2000, p. 15), resulting in class- and race-based discrimination, lack of decision-making power, lack of representation, and unequal access to resources for people of racialized or other marginalized identities. For example, housing options accessible to low-income, racial minorities are more likely to be concentrated in inland flood zones (Ueland and Warf 2006; Chakraborty et al. 2014, p. 8; Collins et al. 2018). Racist practices in the built environment like redlining also contribute to ecological outcomes and patterns, such as reduced tree canopy, reduced species richness, and habitat fragmentation (Kephart 2022, p. 211; Nowak et al. 2022, p. 1; Schell et al. 2020, p. 5–6). This results in greater exposure to increased heat and pollution and reduces opportunities for safe outdoor physical activity. Furthermore, “it is imperative to consider how ongoing colonialism is operating through disasters” (Rivera 2022, p. 133) and, by extension, through buyouts.

Post-disaster recovery and climate adaptation initiatives—such as buyouts—that are rooted in environmental justice can help repair past discrimination, disparities and socio-ecological connections. Addressing the conditions that create social vulnerability in the first place through participatory and collaborative approaches can better meet the needs of low-income people and redress vulnerability to flooding (Bolin and Stanford 1998, p. 35). For example, knowing and caring for the land are both a responsibility and an expression of Indigenous agency for Native Hawaiian communities of Kauaʻi after the 2018 floods (Harangody et al 2022, p. 8). The community-managed stream restoration projects both reduced future flood impacts and aligned with their connection to ʻāina (a fundamental aspect of life, identity, and resilience; a reciprocity between people and place, p. 4). Transforming environmental governance, or the ways in which people collectively make decisions about their surrounding environments, will be key for ecologically sustainable and socially just outcomes in an uncertain world (Salomon et al. 2019; Shi and Moser 2021, p. 5).

Post-disaster or climate-exacerbated contexts reflect a breakdown of socio-ecological relationships and a need for positive repair of these relationships. The above discussion suggests that pathways to repair first require acknowledging that human and environmental well-being constitute each other. Particularly for environmental justice communities, stewardship and care help repair past harm and trauma, build new partnerships with government agencies, and develop positive identities for individuals and their social networks (e.g., Davis and Edge 2022, p. 13; Gordon et al. 2023).

## Floodplain relocation: a breakdown in socio-ecological relationships

In light of the positive socio-ecological connections above, the separation between social and ecological issues in U.S. floodplain buyout programs is both notable and concerning. Most floodplain buyout projects in the U.S. start after a Presidential Disaster Declaration, which allows the federal government to disburse disaster recovery funding. Where buyouts are warranted, state or local governments task an agency—typically in flood control, watershed management, stormwater management, or public works—with implementing the buyout program. Staff in these agencies are responsible for identifying which areas should be prioritized for buyouts and which households are eligible. They conduct property-level outreach to see who is willing to voluntarily participate in the program and organize community meetings in neighborhoods with clusters of buyouts taking place. Once households have agreed to sell their property, programs negotiate the real estate and financial transactions for each household. The property value offered is typically the pre-disaster market value of a home, which may or may not cover the remaining mortgage or allow a homeowner to purchase a comparable home nearby. After the property is purchased, the government demolishes the structures and transfers the deed to a long-term owner, such as the local government or flood control district (Weber and Moore 2019). For many buyout processes, including FEMA’s, this effectively concludes the buyout from the program’s perspective. Whichever local entity assumes property title becomes responsible for envisioning and managing its use and care.

This approach to managing socio-ecological relationships in floodplains reflects the FEMA Hazard Mitigation Grant Program’s overarching goal for post-disaster relocation: to “reduce the risk to individuals and property from natural hazards, while simultaneously reducing reliance on Federal disaster funds” (FEMA 2015, p. 1). These goals—rather than more expansive goals of promoting community and ecological health—shape the program’s design and operations. For instance, to reduce federal fiscal burdens, residents wishing to get a buyout must be located in a community that participates in the National Flood Insurance Program and meet a 1:1 cost–benefit ratio (FEMA 2022; Maryland DEM n.d.).^1^ By contrast, policies do not fund or mandate key elements of social and ecological repair, such as requiring households and businesses to move to a less flood prone area or tracking where they go and how they fare after they move. Nor does the program help restore buyout sites beyond stipulating that they may not be redeveloped.

In most places, staff implementing buyout programs do not have the advantage of institutional memory, as these “pop up” offices interpret regulations, provide support and guidance, and learn the bureaucratic process for the first time. Buyout staff typically make decisions with few resources, mandates, or support to be more creative and integrative in their practices. Building relationships with social and ecologically-oriented organizations, should they not already exist, also requires time. Planning and long-term thinking that advances social and ecological goals, therefore, can be very difficult, if not impossible, to launch in the immediate post-disaster period when people are in need of urgent rehousing or reconstruction.

This policy and organizational framework challenges efforts to design more holistic efforts for restorative community relocation. Below, we discuss how social and ecological processes are deeply entangled both in the traditionally “social” process of relocating residents and the “ecological” process of managing the land. This points to a need to better integrate these components and respond to socio-ecological relationships throughout buyout and restoration processes.

### Socio-ecological relationships impacted by buyout processes

From a government perspective, residents’ relationships with the land end when they sell the property to the government. However, for residents, their relationship with the land rarely concludes with the day they move out. Rather, the ongoing or final state of the property is a significant piece of their story. Conversations with buyout participants and staff who work with them reveal that most people hold a land ethic and personal environmental values. While other factors weigh heavily on their decisions, participants say that knowing the intended environmental stewardship outcomes does make a difference in their willingness to participate.

Many people want reassurance that no one will be harmed or put in danger by being able to rebuild in the same location. They almost always want to know the land will not be sold to someone else to develop, particularly to turn a profit by putting others in harm’s way (workshop with buyout recipients 2022). They want to know that their sacrifice of giving land back to Mother Nature will constitute a legacy (Koslov 2016). It is extremely common for buyout recipients to return to the sites where their homes used to be to observe changes in the land over time (workshop with buyout recipients 2022). If the choice to leave their home resulted in protection for others, a new public asset, or new habitat for plants and animals, they tend to feel a sense of satisfaction and even pride in making those outcomes possible. On the other hand, if the loss of their home resulted in a vacant lot that has contributed to a sense of blight or nuisance in their former neighborhoods, it can sour their experience, even if they don’t regret taking the buyout and the process was otherwise positive. Others who have not yet moved are also watching what happens with buyout sites, and poor care of land will inform their willingness to participate in buyouts in the future.

In addition, place attachment constitutes not just the monetary value of the property, but a sense of identity, belonging, and financial security, the loss of which can contribute to grief and anxiety (Binder et al. 2019; Blaze and Shwalb 2009, p. 319; Greer et al. 2022). The loss of connections to land, place, and community is especially damaging for Indigenous, environmental justice, and other low-income communities whose culture, livelihoods, and ability to survive depend on these place-based connections. Some residents of low-lying areas have diverse and ongoing relationships with the land—from caring for ancestral burial grounds, to food subsistence activities like fishing, hunting, and aquaculture, to maintaining cultural practices related to these activities. These groups also may be particularly attentive to the multi-generational aspects of planning associated with buyouts, or more broadly, with relocation (Jessee 2022; Marino 2018). Buyouts and relocations that sever these relationships can create generational harm.

For Black and brown communities that have experienced generational disinvestment, environmental harms, and displacement, the restrictiveness of buyout programs’ goals and designs can be especially limiting and exclusionary.^2^ For instance, buyout programs’ emphasis on single family dwellings as opposed to more diverse forms of land tenure and building types disproportionately exclude communities of color and low-income groups that live in rental units, co-ops, condos, mobile home parks, or heirs property.^3^ Program design can also exclude collective visions of post-buyout relationships with the land. For instance, the Île de Jean Charles Biloxi-Chitimacha-Choctaw Tribe in Louisiana had envisioned their relocation as an opportunity to reunite tribal members, who had gradually dispersed as they lost land, on communally held land while retaining access to fishing and ancestral sites. However, the Department of Housing and Urban Development managing their relocation funding (from the National Disaster Resilience Competition) focused chiefly on property relocation out of the previous site, without allowing the Tribe to retain access rights, and requiring homes at the new site to be individually-owned (Jessee 2022).

### Socio-ecological relationships impacted by land management post-buyout

Given HMGP’s underlying mission and funding structure, the ecological futures of buyout communities do not feature significantly in the buyout process. Rather, planning what happens with the land often happens after the property has been bought out. As a result, land management strategies post-buyout can be very diverse and divorced from community engagement efforts. At a minimum, the new landowner must provide basic maintenance, such as mowing, to maintain expectations of tidiness, minimize dumping, or prevent illegal activities. In some cases, local governments may turn land over to remaining neighbors and ask them to mow it or use it for gardens. As noted in the introduction, nearly half of sites remain as lawns, lawns with trees, or parking lots, effectively like their former state but without the structures (Zavar and Hagelman 2016, p. 365–8). A few states and localities have created management plans for buyout lands, which can be an important tool for framing goals, indicating the level of engagement with people, and defining the roles and responsibilities for different partners.

The social connection between communities and post-buyout landscapes varies widely depending on the management strategy and the local contexts (Vanucchi et al. in progress). One common challenge of restoring buyout lands is that buyouts often create a checkerboard of vacant properties since FEMA-funded buyouts are voluntary. This can render remaining households more susceptible to perceptions of blight, especially in lower-income and minority communities, which makes them especially sensitive about how buyout sites are managed. In Austin, for instance, city staff would prefer restoring some buyout sites to prairie grasses, but remaining residents are adamant about continuous mowing. Staff recognize that their concerns reflect not only worries about the site, but deeper issues of government mistrust, fear of disinvestment, and lack of greater awareness of the community benefits of restoration (workshop with buyout managers, 2021).

In some of the biggest buyout programs that have amassed sizable land and have financial resources, post-buyout land management can have limited social engagement with residents or efforts to deliver socially-beneficial projects. This disconnected approach contrasts sharply with the deeply engaged process of negotiating the buyout. This limited engagement is especially true where management plans prioritize biodiversity or hydrological function. For example, the Harris County Flood Control District serving Houston, Texas, maintains buyout sites to minimize vegetation (such as trees or tall grasses) and maximize flood storage. Here, green infrastructure plays a similar role as gray infrastructure and has limited social uses. In Washington State, the buyout program prioritizes salmon habitat restoration by removing levees and restoring habitat to reconnect waterways. The program works extensively with Tribes, farmers, and conservation groups to deliver these projects, which prioritize biodiversity metrics but not social outcomes.

Finally, a few places have effectively restored urban buyout lands and delivered beneficial social and ecological outcomes. The buyout program in Austin, Texas, for example, emphasizes both ecological restoration and community well-being. Their community visioning and master planning process for Williamson Creek involved local nonprofits and led to a project to create a creekside greenway for a low-income community of color (Kodis et al. 2021). Land managers in places like Charlotte-Mecklenburg, North Carolina, have invested heavily in restoring some buyout sites to create local greenways, as prioritized by local communities. However, these efforts (coupled with the area’s hot housing market) have accelerated gentrification in some neighborhoods (Interviews with Charlotte-Mecklenburg Stormwater Services staff, 2021). This means that the original residents might be priced out and that fewer lower-income people can afford to live in a location with new natural amenities.

Across these experiences, programmatic support for community-driven land management reflects the lack of integrated or available funding across buyouts and ecological restoration. This significant constraint makes it hard to plan for future land management with potential buyout participants. Only recently has FEMA begun to allow biodiversity and ecological conservation criteria to be considered as part of the overall cost–benefit analysis, but buyout funding continues to fund only the purchase of the home (Kodis et al. 2021). Moreover, many small local governments have limited funds for buyout site management. Often, this is because disasters negatively impact revenue, as buyouts reduce property tax rolls while expenditures remain constant in checkerboarded neighborhoods (BenDor et al. 2020; Curran-Groome et al. 2022). Some municipalities, therefore, seek out additional grant or foundation funding, or collaborate with a conservation organization, and sometimes transfer title and management responsibilities to a land trust (Kodis et al. 2021). Conservation organizations themselves have historically prioritized areas with high existing biodiversity rather than urbanized land (conversations with staff from The Nature Conservancy and Environmental Defense Fund, 2021, 2022). Local government staff and conservation organizations alike are confronting a new frontier in land management. Their struggles can limit efforts to restore the land at all, much less include communities in the process.

### Breakdown in socio-ecological relations in floodplains

As residents’ and managers’ experiences demonstrate, the buyout process is deeply intertwined with people’s relationships with place and land, while land management raises a multitude of issues around social relations. Yet, the focus in many buyout programs is on either compensating owners for their property value and managing the land’s ecological or hydrological function rather than respecting these locations as places with which people have relationships of memory, subsistence, or community. The current dynamics reflect a reductive approach to property rather than an expansive approach to land relations (Nedelsky 2022). This reductive approach denigrates alternatives that already exist and limits our imagination of more restorative and reparative approaches that are meaningful to those who leave and, at a minimum, not damaging to those who stay.

**Fig. 2:**
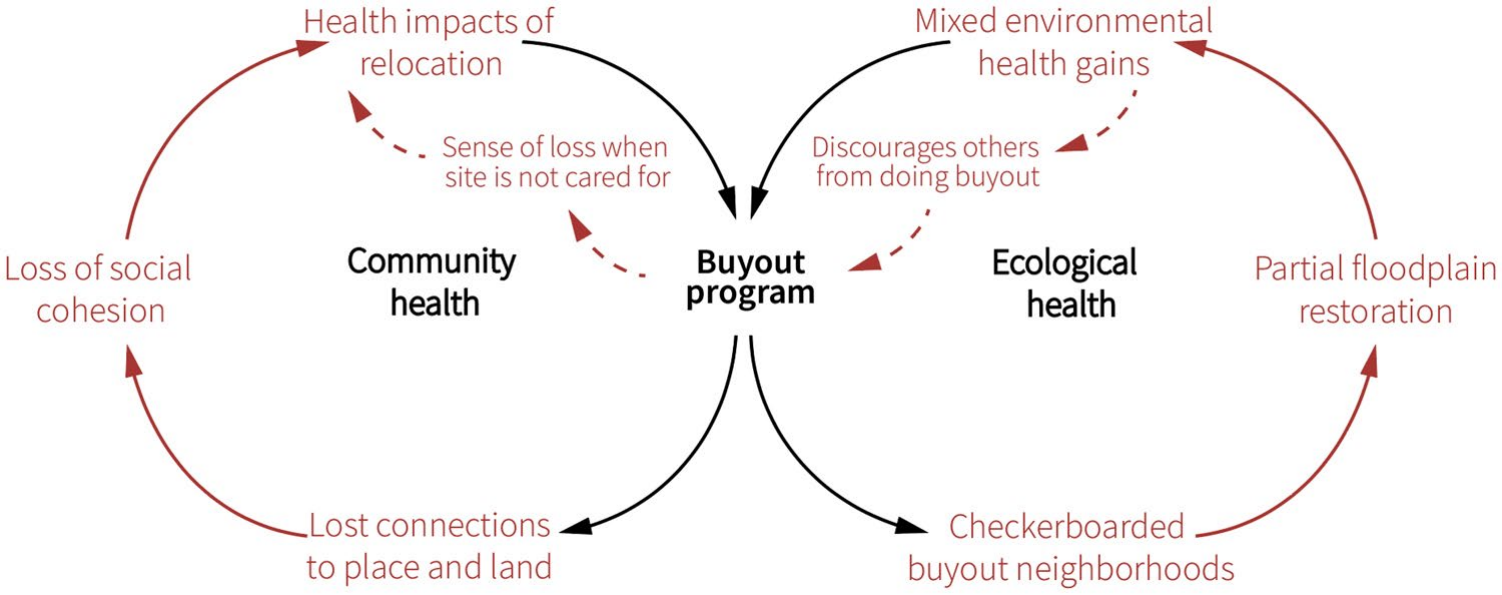
Divisions in Floodplain Buyout Programs’ Socio-Ecological Relationships

In Fig. 2, we posit that buyout processes can negatively affect socio-ecological relationships in post-disaster contexts. The circle on the left focuses on community health, showing how buyouts can sometimes sever people’s connections to place and land, erode social cohesion, and limit health or human flourishing. The circle on the right focuses on ecological health, showing how buyouts can sometimes produce checkerboarded landscapes that can inhibit floodplain restoration, and result in mixed ecological outcomes. Neglect or lack of site restoration can contribute to a sense of loss among buyouts participants and discourage others from participating in buyouts in the future.

## Bridging socio-ecological divides in floodplain relocation

The juxtaposition of the buyout process against examples of positive socio-ecological land relations suggests that there are opportunities to improve buyout programs. As this essay suggests, people living on the frontlines of flooding yearn for relocation programs that recognize:

- The historic and ongoing harms their communities have experienced,
- The centrality of place-based community to their daily functioning and well being,
- People’s diverse ways of owning or living in relation to the land,
- The opportunity for a trauma-informed approach to buyouts, and
- The regenerative potential of the land and people’s aspirations to leave a legacy after they move.

These desires reflect a concern for human, community, and ecosystems health and a culture of care to achieve conditions that allow these groups to flourish (Carr 2022).

Below, we describe three general strategies to help buyout programs and neighborhoods build such a culture of care in response to the trauma and grief associated with disasters, land loss, and relocation. Many practical and systemic barriers inhibit better marrying social and ecological factors in floodplain buyout processes and outcomes. It is, therefore, with humility that we suggest the following, with full recognition that the people working in these spaces and the residents faced with these difficult decisions are already overwhelmed and that buyouts are under-resourced and constrained in many ways. Our hope is that these suggestions are met with creativity and openness to spark new conversations.

### Create enabling conditions for equity and stewardship

Sustaining socio-ecological approaches to floodplain adaptation requires reforming existing institutions and developing new institutional capacity. Federal agencies should not only fund capital projects, but also institution building at state and regional levels to implement buyout and climate mobility projects (Shi et al. 2022). In addition, federal buyout and restoration programs can better incorporate socio-ecological concepts. For instance, the rollout of President Biden’s Nature-Based Solutions Roadmap (2022), 2022 Inflation Reduction Act funding, and FEMA’s growing Building Resilient Infrastructure and Communities (BRIC) and HMGP programs all provide opportunities to incorporate ecological resilience and community well-being into their goals and evaluation criteria. Making it easier for states and localities to mix buyout and restoration funding would also enable projects to adopt strategies that promote both social and ecological outcomes.

### Create more inclusive and coordinated planning and engagement processes

Buyout programs can better enable socio-ecologically integrative projects by including more diverse participants early on in the planning process. This includes drawing on a broader array of professions (including housing and social services), working with planners as boundary spanners, and involving communities—especially Black, Hispanic, and Indigenous ones—in designing and setting the parameters of relocation and future land use. Governments can develop clearer coordinating mechanisms between buyout and land managers if they do not yet exist. Understanding and respecting each other’s roles, responsibilities, constraints, and pressures is a step toward aligning plans and priorities. Planners, who have historically not been involved in buyout planning, can also help play an integrative role in supporting collaboration (Innes and Booher 2018). In truth, anyone with an interest in the outcomes and is respected in the community can convene people with a range of perspectives to attempt to address relocation challenges together. Tribes, frontline communities, watershed associations, community land trusts, housing advocates, or land stewardship groups represent some of the critical voices. Buyout program staff should not only share information about what will happen with the land after the buyout but also work with residents to jointly develop principles, if not plans, for post-disaster recovery, buyouts, and restoration. Communications should take care to make outreach as inclusive and supportive as possible, for instance by using multiple languages, contacting people repeatedly, and being comprehensible to people with lower literacy levels.

### Reframe and broaden floodplain adaptation goals

A more inclusive process can help buyout programs reframe goals in management plans to encompass the health and justice dimensions noted above. Broader goals and strategies could include steering relocatees towards higher elevation rehousing, addressing mental health and spiritual needs related to place-based attachment, restoring or maintaining lands for desired community uses, and providing opportunities for both those who have moved and those who are staying to help steward the land. These steps can help increase buyout participation, improve resident mental and physical health, and diversify who can access buyout lands and how. Involving residents in potential buyout neighborhoods early on can help reduce anxiety and build a constituency of support pre-and post-buyout so that relocation becomes less traumatic and more reflective of desired changes and healing. This, in turn, can create reduced obligations on local service maintenance and more opportunities for restoring contiguous areas. Pluralizing post-buyout land management strategies can break down traditional framings of land as being either developed or conserved and support more sustainable land relations. Programs can then broaden their metrics of success to reflect more plural approaches to human and ecological health and equity (Atoba et al. 2021; Kraan et al. 2021).

**Fig. 3:**
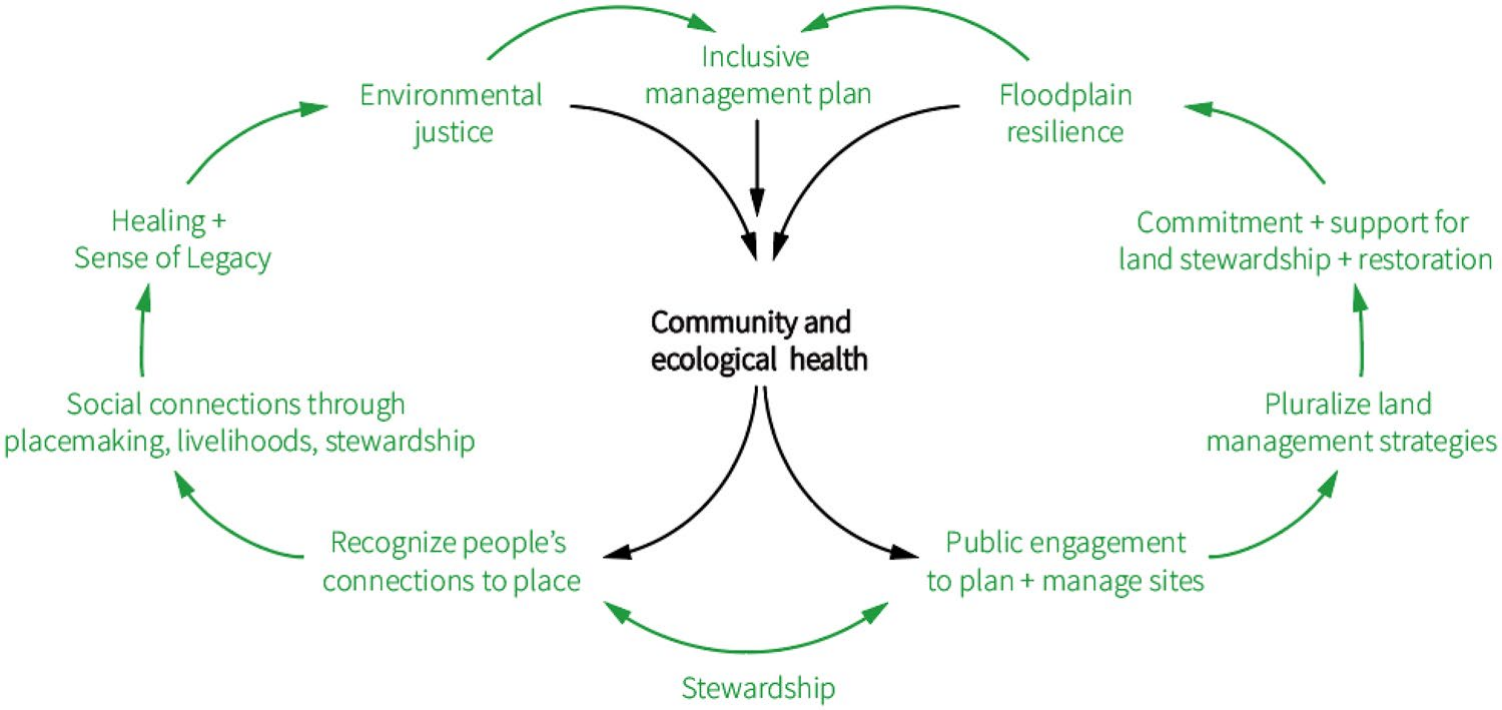
Benefits of Virtuous Socio-Ecological Relationships in the Floodplain

Together, these practices can help bring more positive socio-ecological dynamics to efforts to relocate from floodplains. Figure 3 shows the positive reinforcing relationships between human and ecological health in floodplains. On the left, recognizing people’s connections to place supports continued opportunities to engage in placemaking, livelihoods, and stewardship. This in turn contributes to a sense of legacy, healing, and environmental justice for those most disadvantaged by relocation. Increasing participation can create more contiguous buyout lands that enable restoration projects. On the right, efforts to engage residents and the public in planning and managing the future use of these sites can lead to more plural land management strategies that secure greater commitment and support for ongoing land stewardship and restoration. Building and institutionalizing a culture of caring land relations in buyout programs can improve the way buyout programs are perceived and, therefore, supported. These steps will not eliminate the sense of loss, grief, and trauma due to climate change and relocation (Tschakert et al. 2017). Nor will the benefits of restoration equally accrue to all those impacted by relocation projects. Nevertheless, any steps to improve socio-ecological ties is better than the status quo, and is likely to provide direct and indirect benefits for many.

## Conclusion

In an era of climate change, vast geographies are undergoing ecological and social transition. This creates existential questions for the human and non-human communities that currently inhabit these areas. Who will occupy these areas in the future and how? How can existing governance strategies evolve to better enable socially and ecologically restorative adaptation in these places? We have argued that a more holistic perspective of floodplain relocation—one that refuses to separate social from ecological considerations—is both helpful and necessary for the future evolution of flood-prone communities.

Such a physical and governance transition is not without structural, operational, and conceptual challenges. While we put forward potential strategies to advance more socially and ecologically intertwined practices, we also recognize that there are many empirical gaps in the science underlying more innovative approaches. There is an urgent need for research to answer questions like, how much do past or proposed ecological outcomes affect people’s decisions to relocate? Do places with more socially progressive relocation programs result in better ecological outcomes? To what extent does the environmental condition of post-relocation landscapes (as well as opportunities for stewardship) affect residents, both those who have moved and those that remain behind? When, how, and why are historically marginalized people included in the decision-making process? What kinds of government regulations prohibit or enable more integrated socio-ecological approaches to floodplain relocation, whether in the United States or internationally? What factors make those policies or outcomes possible? Such questions can be difficult to answer because implemented programs rarely integrate social and ecological components. This suggests that scholars have an opportunity to work alongside practitioners in designing and measuring policy experimentation on the ground.

Relocation efforts like buyouts can be transformative. However, the real transformation lies not just in how a site looks and functions, but in how we govern the transition process. To date, relocation efforts have often been conceived of on a disaster-by-disaster basis, segmenting housing relocation from land management. The governance structure supporting this approach is becoming increasingly untenable as climate change increases the frequency and reach of flooding. In a broader context, the social and ecological divides seen in relocation efforts (not just in the U.S., but around the world) reflect the pervasive modern development paradigm of separating conserved areas from developable areas. Seen this way, recognizing people’s land ethics in decisions to relocate, involving local communities in planning and caring for post-buyout areas, and prioritizing human and ecological health metrics in this process is itself a radical political project. A socio-ecologically integrated approach can improve existing programmatic efforts, and even open conversations and pathways to more transformative change.

## Footnotes

1 This means that the cost of purchasing and demolishing the property must be less than or equal to the cost of future recovery and restoration of the property due to flooding. These standards are changing for low-income communities. In October 2022, FEMA announced an alternative cost-effectiveness methodology (for its Building Resilient Infrastructure and Communities (BRIC) and Flood Mitigation Assistance (FMA) Grant Programs) with the goal of supporting impoverished, Tribal, or socially vulnerable communities (FEMA 2022).

2 Depending on when, where, and how community meetings and announcements are phrased, what language they are written in, who distributes them and the timing and location of the meetings, this process in and of itself can lead to exclusion or prioritization of certain neighborhoods or people.

3 Heirs property is left to family members without a formal will or registration of title change. Holders of heirs property are disproportionately African-American, due to lack of access to lawyers and supportive systems for transferring property. They have long had difficulty establishing claims on buyout funding, but FEMA revised the documentation requirements for buyouts in 2022 to make it more accessible (FEMA 2022).
